# Galactose-Modified PH-Sensitive Niosomes for Controlled Release and Hepatocellular Carcinoma Target Delivery of Tanshinone IIA

**DOI:** 10.1208/s12249-021-01973-4

**Published:** 2021-03-10

**Authors:** Xixi Hu, Jun Zhang, Lulu Deng, Hao Hu, Junjie Hu, Guohua Zheng

**Affiliations:** 1 School of Pharmacy, Hubei University of Chinese Medicine, No. 1, West Huangjiahu Road, Wuhan, 430065 People’s Republic of China; 2 Department of Pharmacy, Xiangyang No. 1 People’s Hospital, Xiangyang, People’s Republic of China; 3 Department of Pharmacy, Yichang Special Care Hospital, Yichang, People’s Republic of China; 4 Department of Orthopedics, People’s Hospital of Yuanan, Yuanan, People’s Republic of China; 5 Key Laboratory of Chinese Medicine Resource and Compound Prescription, Ministry of Education, Hubei University of Chinese Medicine, No. 1, West Huangjiahu Road, Wuhan, 430065 People’s Republic of China

**Keywords:** Galactose modification, Niosomes, pH sensitivity, Tanshinone IIA, Targeting ability

## Abstract

Increasing the drug tumor-specific accumulation and controlling their release is considered one of the most effective ways to increase the efficacy of drugs. Here, we developed a vesicle system that can target hepatoma and release drugs rapidly within tumor cells. This non-ionic surfactant vesicle is biodegradable. Galactosylated stearate has been used to glycosylate the vesicles to achieve liver targeting; replacement of a portion (Chol:CHEMS = 1:1) of cholesterol by cholesteryl hemisuccinate (CHEMS) allows for a rapid release of drugs in an acidic environment. *In vitro* release experiments confirmed that galactose-modified pH-sensitive niosomes loaded with tanshinone IIA had excellent drug release performance in acid medium. *In vitro* experiments using ovarian cancer cells (A2780), colon cancer cells (HCT8), and hepatoma cell (Huh7, HepG2) confirmed that the preparation had specific targeting ability to hepatoma cells compared with free drugs, and this ability was dependent on the galactose content. Furthermore, the preparation also had a more substantial inhibitory effect on tumor cells, and subsequent apoptosis assays and cell cycle analyses further confirmed its enhanced anti-tumor effect. Results of pharmacokinetic experiments confirmed that the vesicle system could significantly extend the blood circulation time of tanshinone IIA, and the larger area under the curve indicated that the preparation had a better drug effect. Thus, the results of biodistribution experiments confirmed the *in vivo* liver targeting ability of this preparation. Niosomes designed in this manner are expected to be a safe and effective drug delivery system for liver cancer therapy.

## INTRODUCTION

Liver cancer is a type of malignant tumor that threatens human health. From the perspective of histopathology, more than 90% of liver cancer is of the hepatocellular carcinoma (HCC) type. Liver cancer has a high mortality rate, especially among male patients. According to the International Agency for Research on Cancer (IARC) in 2018, the mortality rate of male patients with liver cancer reached 10.2%. At present, several potential chemotherapeutic drugs that can treat HCC have toxic side effects and low specificity (1), which is the direct reason for their limited use or dosage. Therefore, it is a challenge to provide anti-HCC drugs with low toxicity and high efficacy.

Niosomes, also known as non-ionic surfactant vesicles (NSVs), are single-layer or multi-layer drug carriers that are formed by non-ionic surfactants and cholesterol and belong to the nanoparticles dispersion system. When amphiphilic non-ionic surfactants are dispersed in the water phase, the hydrophobic tails of the molecules tend to clump together to avoid the water phase, while the hydrophilic head is exposed to the water phase, thereby forming closed vesicles with a bimolecular layer structure that can accommodate molecules of different solubilities (2). Niosomes can increase stability, improve efficacy, reduce toxic and side effects of the drug, and prolong the duration of drug action (3). In addition, niosomes also have characteristics of biodegradability and low cost of use due to the excellent biocompatibility and non-oxidizable properties of the non-ionic surfactant (4).

Tanshinone IIA (TanIIA), a quinone compound, is a representative of the fat-soluble components of Salvia miltiorrhiza Bge (5) (molecular formula: C_19_H_18_O_3_), which has a potent effect on inducing apoptosis and broad-spectrum anticancer activity (6–8). Due to the insolubility in water and the short half-life of tanshinone IIA (9, 10), its clinical use is greatly limited (11). Thus, encapsulation of tanshinone IIA by using niosomes can improve its physicochemical properties and improve its clinical application prospect (12, 13).

In order to encapsulate tanshinone IIA by niosomes and to enhance their anti-hepatocellular carcinoma effects, we aimed to increase the concentration in the liver to fully release tanshinone IIA in tumor cells. This requires the vesicles to be “smart,” so they can identify the liver site and have a unique ability to “sense” tumor cells. To enhance the ability to localize and control drug release (14), niosomes are modified with galactose to specifically target them to the liver (15), and pH-sensitive excipients are added to the niosomes to control drug release at the tumor site (16).

We aimed to design a low-molecular-weight lipid named galactosylated stearate (Gal) as a modification of niosomes. The structure of this ligand is similar to the formation of non-ionic surfactants with a water-soluble head and a fat-soluble tail, thus it can be orderly arranged in the bimolecular layer (17). Galactose-modified niosomes are prepared using this ligand, and the fat-soluble tails (usually long-chain fatty acids) are “arranged orderly” in the bimolecular layer of non-ionic surfactants, while the water-soluble galactose heads tend to face the side of the aqueous phase. The water-soluble head is exposed to the surface of the bimolecular layer and can be recognized specifically by the asialoglycoprotein receptor (ASGPR) on the hepatocyte membrane, which can mediate hepatocyte endocytosis, thereby internalizing galactosylated macromolecules within the cells (18).

CHEMS (pH), a cholesterol derivative, is a pH-sensitive excipient. Like cholesterol, CHEMS also has natural lipophilicity and membrane stabilization activity (19, 20). CHEMS self-assemble into a bimolecular layer in alkaline and neutral aqueous media and therefore fuse with the bimolecular layer of niosomes, whereas they rupture the bimolecular layer in acidic media to facilitating drug release (21). Intracellular and extracellular acidity has been recognized as a common feature of tumor tissue (22, 23), and the intracellular acidity is the stronger characteristic (24–26). When niosomes containing CHEMS encounter tumor cells, the bimolecular layer becomes unstable due to the environmental acidity, thus promoting release of the payload (27).

Based on the above-mentioned theory, galactose-modified pH-sensitive niosomes loaded with tanshinone IIA were prepared by an ethanol injection method. The pH sensitivity of the preparation was investigated via *in vitro* release experiments, and targeting performance of the preparation was studied by cell uptake experiments. The inhibitory effect of the preparation on tumors was tested by the CCK8 assay, and the anti-tumor effect of the preparation was analyzed further by apoptosis and cell cycle analyses. Finally, the *in vivo* process of the drug was preliminarily evaluated by pharmacokinetic experiments and analyses of pharmacokinetic parameters. Taken together, we found that this new drug delivery system is promising for the treatment of HCC.

## MATERIALS AND METHODS

### Materials

Tanshinone IIA was purchased from Chengdu Push Biotechnology Co., Ltd. (Chengdu, China), with a purity of 99.71%, and was determined by liquid chromatography at 254 nm (Chengdu, China). Galactosylated stearate was produced by Xi’an Ruixi Biotechnology Co., Ltd. (Xi'an, China). Cholesteryl hemisuccinate was purchased from Xi’an Ruixi Biotechnology Co., Ltd. (Xi’an, China). Cholesterol was purchased from Beijing Solarbio Science & Technology Co., Ltd. (Beijing, China). Span80 was purchased from Beijing Solarbio Science & Technology Co., Ltd. (Beijing, China). Nile Red was purchased from Beijing Solarbio Science & Technology Co., Ltd. (Beijing, China). Phosphate- buffered saline (PBS) was purchased from HyClone (Logan, UT, USA). Fetal bovine serum (FBS) was purchased from HyClone (Logan, UT, USA). Dulbecco’s modified Eagle’s medium (DMEM) was purchased from HyClone (Logan, UT, USA). Chromatographic-grade acetonitrile was purchased from Sigma Aldrich (St. Louis, MO, USA). Chromatographic-grade methanol was purchased from Sigma Aldrich (St. Louis, MO, USA). The Cell Counting Kit-8 (CCK8) was purchased from Dojindo Laboratories (Kumamoto, Japan). Cell Apoptosis and Cycle Analysis Kit were purchased from Nanjing Biobox Biotechnology Co., Ltd. (Nanjing, China). Other chemicals and reagents were of analytical grade. Tumor cell lines (HepG2, Huh7, A2780, and HCT8) were obtained from the Shanghai Institute of Biochemistry and Cell Biology, Chinese Academy of Sciences (Shanghai, China). Plasmids used for transfection, including pT3-EF1α-HA-myr-AKT, pT3-EF1α-V5-c-Met, and pCMV/sleeping beauty transposase (SB) were supplied by Professor Xin Chen’s laboratory at the University of California (San Francisco, CA, USA).
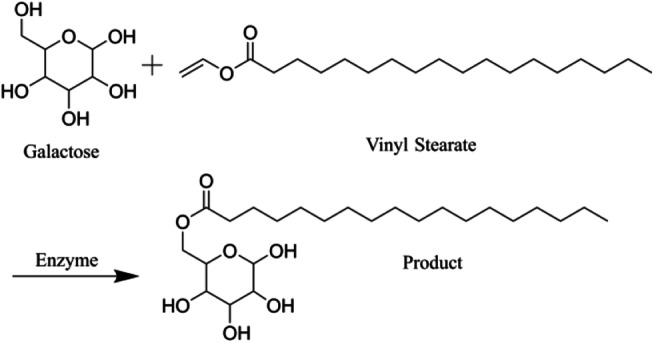


We proposed to produce a type of asialoglycoprotein ligand modifier that can be linked to the surface of vesicles by esterification of galactose and vinyl stearate with lipase (Novozym 435). The reaction formula is shown in the figure above. Considering the purification and yield of the product, we commissioned Xi’an Ruixi Biotechnology Co., Ltd. to produce galactosylated stearate.

### Animals

Wild-type friend leukemia virus B/NIH (FVB/N) mice were purchased from Beijing Vital River Laboratory Animal Technology Co., Ltd. (Beijing, China). Sprague-Dawley (SD) rats were purchased from Liaoning Changsheng Biotechnology Co., Ltd. (Benxi, China). Research behavior studies for all laboratory animals were approved by the Animal Ethics Committee of Hubei University of Chinese Medicine (Wuhan, China) and followed the standard set forth in the eighth edition of the *Guide for the Care and Use of Laboratory Animals* published by the Shanghai Scientific & Technical Publishers (Shanghai, China).

### Preparation of Galactose-Modified PH-Sensitive Tanshinone IIA-Loaded Niosomes

Taking the encapsulation efficiency as an index, representative factors (dosage of tanshinone IIA, Span80, and cholesterol or CHEMS, stirring speed, hydration temperature) were selected through a single-factor examination to design an orthogonal experiment, and the optimal formulation and process of the preparation was determined based on the experimental results. The precisely weighed prescription amounts of tanshinone IIA, Span80, cholesterol, CHEMS, and galactosylated stearate (molar ratio, 1:30:15:15:6) were placed in a 50-mL beaker and fully dissolved with 5 mL of ethanol (28). The ethanol solution was slowly and uniformly injected with a syringe into 15 mL PBS (pH 7.38) at a constant temperature of 60°C and stirred at 600 r/min (3). The solution was stirred for a specific time until the ethanol was entirely evaporated, resulting in a final volume of 10 mL. After the temperature of the concentrated hydrate reached room temperature, the solution was homogenized by ultrasonic cell crusher for 10 min (7). Next, the particle size of the vesicles was reduced by a liposome extruder (*MORGEC LE-15, USA*). Finally, the extruded solution was placed in a sterilized centrifuge tube to obtain targeted niosomes loaded with tanshinone IIA. Targeted niosomes loaded with tanshinone IIA are abbreviated as follows: galactose modification (Gal)-pH sensitivity (pH)-tanshinone IIA (TanIIA)-simple nonionic surfactant vesicles (NSVs).

Mentioned above is the final form of the prepared drug containing vesicles; however, during the experimental studies, we prepared vesicles of several other transition morphologies, including TanIIA-NSVs, Gal-TanIIA-NSVs, and pH-TanIIA-NSVs. These vesicles were all prepared by the ethanol injection method and varied in individual composition. TanIIA-NSVs were prepared from tanshinone IIA, Span80, and cholesterol (molar ratio, 1:30:30). Gal-TanIIA-NSVs were prepared from tanshinone IIA, Span80, cholesterol, and galactosylated stearate (molar ratio, 1:30:30:6), and pH-TanIIA-NSVs were prepared from tanshinone IIA, Span80, cholesterol, and CHEMS (molar ratio, 1:30:15:15).

### Characterization of Galactose-Modified PH-Sensitive Tanshinone IIA-Loaded Niosomes

#### Transmission Electron Microscopy

Targeted niosomes loaded with tanshinone IIA were morphologically characterized by transmission electron microscopy (TEM) (JEOL JEM-1400, JPN). The copper mesh was immersed in a sample cell filled with niosomes suspension that was prepared according to the above-mentioned method, then taken out and placed on filter paper for several minutes to absorb excess liquid. The phosphotungstic acid solution was dropped onto the copper mesh to stain the sample, which was used for TEM observation after the sample was dried.

#### Nanoparticle Size and Zeta Potential Analyzer

The particle size, distribution, and potential of targeted niosomes loaded with tanshinone IIA were observed by a nanoparticle size and zeta potential analyzer (Malvern Nano-ZS90, GBR). The prepared niosomes were directly loaded into the sample cell for particle size and potential determination after extrusion through a liposome extruder. Each parameter was measured in triplicate at 25°C.

#### Encapsulation Efficiency

The encapsulation efficiency of targeted niosomes loaded with tanshinone IIA was determined by high-performance liquid chromatography (HPLC). The chromatographic conditions were as follows: InertSustain C18 (column length: 150 mm, inner diameter: 4.6 mm, particle size: 5 μm), acetonitrile-water (67:33) as mobile phase, detection wavelength: 270 nm, column temperature: 20°C, flow rate: 0.8 mL/min (5). The encapsulation efficiency of the vesicles was determined by low-speed centrifugation (29). After the niosomes solution was centrifuged at low speed (69.53×*g*), unencapsulated drug precipitates on the inner wall of the centrifuge tube and the remaining fraction represented the amount of drug encapsulated in the preparation, and the amount of drug in the non-centrifuged niosomes solution represented the total amount of drug encapsulated and unencapsulated in the preparation. The encapsulation efficiency of the vesicles was calculated by comparing the drug amount before and after centrifugation. The encapsulation efficiency (EE) was calculated as follows (30):

$$\mathrm{EE}\left(\%\right)=\frac{\begin{array}{c}\mathrm{Amount}\ \mathrm{of}\ \mathrm{drug}\ \mathrm{encapsulated}\ \mathrm{in}\\ {}\ \mathrm{the}\ \mathrm{microparticle}\ \mathrm{preparations}\end{array}}{\begin{array}{c}\mathrm{Total}\ \mathrm{amount}\ \mathrm{of}\ \mathrm{encapsulated}\\ {}\ \mathrm{and}\ \mathrm{unencapsulated}\ \mathrm{drug}\ \mathrm{in}\\ {}\mathrm{the}\ \mathrm{microparticle}\ \mathrm{preparations}\end{array}}\times 100\%$$

#### Leakage

Prepared vesicles were put into a vial; sealed vials were protected from light and stored at 4°C. Vesicles were inspected for leakage for the next 60 days. The encapsulation efficiency during different storage periods was calculated according to the above-mentioned method, and leakage was expressed by the change of encapsulation efficiency during storage. Leakage was checked when the nanoparticles preparation product was dispersed in a liquid medium for storage. Leakage was calculated as follows (30):

$$\mathrm{Leakage}=\frac{\begin{array}{c}\mathrm{Amount}\ \mathrm{of}\ \mathrm{drug}\ \mathrm{encapsulated}\ \\ {}\mathrm{before}\ \mathrm{storage}-\mathrm{amount}\ \mathrm{of}\\ {}\ \mathrm{drug}\ \mathrm{encapsulated}\ \mathrm{after}\ \mathrm{a}\\ {}\mathrm{certain}\ \mathrm{period}\ \mathrm{of}\ \mathrm{storage}\end{array}}{\begin{array}{c}\mathrm{Amount}\ \mathrm{of}\ \mathrm{drug}\ \\ {}\mathrm{encapsulated}\ \mathrm{before}\ \mathrm{storage}\end{array}}\times 100\%$$

### PH-Sensitive Property Studied by *In Vitro* Release Experiments

A total of 3 mL of tanshinone IIA reference solution (Free TanIIA), galactose-modified tanshinone IIA-loaded niosomes (Gal-TanIIA-NSVs), and galactose-modified pH-sensitive niosomes loaded with tanshinone IIA (Gal-pH-TanIIA-NSVs) were measured and placed in their respective MD44 dialysis bags (MW: 8000~14,000). Dialysis bags were placed in 30 mL release medium containing 10% ethanol at pH 7.38, 6.81, and 5.60, respectively. *In vitro* release experiments were carried out by a constant temperature oscillation method (37°C, 100 r/min), and 1 mL of release medium was quantitatively measured out at a specific time point. Release medium with the same volume and temperature was immediately supplemented to ensure that the dialysis environment was unchanged, and the drug concentration in each release medium was determined. The accumulative release percentage (*Q*) was calculated as follows, and release curves were plotted (31).

$${Q}_{\mathrm{release}}=\frac{\left({C}_1+{C}_2+\cdots +{C}_{n-1}\right)\bullet {V}_1+{C}_n\bullet {V}_2}{m}\times 100\%$$

In the formula, *C*_*n*_ is the sample concentration after sampling at each time point, *m* is the labeled amount of the preparation, *V*_1_ is the fixed sampling volume at each time point, and *V*_2_ is the volume of the dissolution medium.

### *In Vitro* Targeting Studied by Cell Uptake Assay

### Qualitative Investigation of Targeting in Vitro by Inverted Fluorescence Microscopy

Nile Red (NR) was used instead of tanshinone IIA to prepare Nile Red-labeled targeted niosomes with galactosylated stearate dosages of 0, 5%, 10%, 15%, and 20%, respectively (0% Gal-pH-NR-NSVs, 5% Gal-pH-NR-NSVs, 10% Gal-pH-NR-NSVs, 15% Gal-pH-NR-NSVs, 20% Gal-pH-NR-NSVs). A2780, HCT8, Huh7, and HepG2 cells were treated with medium-diluted 10% Gal-pH-NR-NSVs for 4 h, respectively. Treated cells were washed three times with PBS, and fluorescence photographs were taken using an inverted fluorescence microscope to qualitatively analyze the cellular uptake of niosomes.

### Quantitative Investigation of Targeting *In Vitro* by Flow Cytometry

Huh7 and HepG2 cells were treated with medium-diluted niosomes with a galactosylated stearate dosage of 0, 5%, 10%, 15%, 20%, and free NR solution at an equivalent NR dose of 20 μg/mL for 4 h, respectively. Similarly, A2780, HCT8, Huh7, and HepG2 cells were treated with 10% Gal-pH-NR-NSVs to verify the results of the qualitative experiments. After incubation, adherent cells were washed three times with PBS, digested with trypsin-EDTA solution, and digested cells were transferred to centrifuge tubes. Cells were centrifuged at 123.8×*g* for 5 min, and the supernatant was discarded. Then, cells were gently resuspended with PBS and counted. A certain amount of resuspended cells was centrifuged at 123.8×*g* for 5 min (32), the supernatant was discarded, and 500 μL of PBS was added to resuspend the cells, then flow cytometry was performed.

### Cell Viability Determined by the CCK8 Assay

#### Determination of Cytotoxicity of Niosomes Materials

A2780, HCT8, HepG2, and Huh7 cells were seeded in 96-well plates at a density of 4 × 10^4^ cells per well for 24 h (32) and treated with complete medium containing a series of blank niosomes (NSVs, Gal-NSVs, pH-NSVs, Gal-pH-NSVs) for 24 h. Subsequently, the culture medium was discarded, and CCK8 solution was added to the cells. Then, cells were continuously cultured for 2 h at 37°C. The absorbance value of each well was measured at 450 nm by a microplate spectrophotometer (BIO-RAD xMark, USA). The control group and blank group were included in the experiment. The control group included cells that did not receive drugs, and the blank group has neither cells nor drugs. Cell viability was calculated according to the following formula (33):

$$\mathrm{Cell}\ \mathrm{viability}\ \left(\%\right)=\frac{A_{\mathrm{test}}-{A}_o}{A_{\mathrm{contrast}}-{A}_o}\times 100\%$$

In the formula, *A*_test_ is the absorbance value of the test group, *A*_contrast_ is the absorbance value of the control group, and *A*_0_ is the absorbance value of the blank group.

#### Inhibitory Effects of Tanshinone IIA and Tanshinone IIA-Loaded Niosome on Tumor Cells

A2780, HCT8, HepG2, and Huh7 cells were seeded in 96-well plates for 24 h and treated with serial concentrations of tanshinone IIA solution (Free TanIIA) and tanshinone IIA-loaded niosomes (TanIIA-NSVs, Gal-TanIIA-NSVs, pH-TanIIA-NSVs, Gal-pH-TanIIA-NSVs) at an equivalent tanshinone IIA dose for 24 h. Then, the culture medium was discarded, CCK8 solution was added to the cells, and cells were continuously cultured for 2 h at 37°C. The absorbance value of each well was measured at 450 nm by a microplate spectrophotometer (33).

### Cell Apoptosis Determined by Flow Cytometry

HepG2 cells were seeded in 6-well plates at a density of 2 × 10^5^ cells per well for 24 h and treated with tanshinone IIA solution (Free TanIIA) and tanshinone IIA-loaded niosomes (TanIIA-NSVs, Gal-pH-TanIIA-NSVs) at an equivalent tanshinone IIA dose of 5 μg/mL for 24 h. Another group was treated with PBS for the same duration as the control group. After incubation, the cell culture medium was transferred to a 2-mL centrifuge tube, and cells digested by a trypsin-EDTA solution were added to the cell culture medium collected previously. Subsequently, the cells were mixed, transferred to a centrifuge tube, and centrifuged at 1000×*g* for 5 min (34). The supernatant was discarded, and the cells were gently resuspended with PBS and counted. A certain amount of resuspended cells were centrifuged in a similar fashion, the supernatant was discarded (PBS was removed), and 500 μL of binding buffer was added. Then, the cells were gently resuspended, and 5 μL Annexin V-FITC was added. After mixing, 5 μL propidium iodide (PI) staining solution was added to the cells, gently mixed, and incubated for 10 min in the dark at room temperature (20–25°C). Flow cytometry was then performed (35).

### Cell Cycle Determined by Flow Cytometry

The test method was performed according to the method provided with the apoptosis test until two centrifugations were completed, and the supernatant was discarded. Next, 1 mL of ice bath precooled 70% ethanol was added to fix the cells for 24 h at 4°C, then, the fixed cells were centrifuged at 1000×*g* for 5 min (36). After resuspending the cells, the cells were centrifuged and precipitated again, and the supernatant was discarded. A total of 500 μL PI staining solution was added to the cells, and the cells were slowly and sufficiently resuspended and bathed for 30 min in the dark at 37°C. Flow cytometry was completed within 24 h after staining (37).

### Pharmacokinetics and Biodistribution Studies of Tanshinone IIA and Tanshinone IIA-Loaded Niosomes

Twelve male SD rats (weighing 200–250 g) were randomly divided into three groups. Rats were fasted for 12 h but drank water freely before administration (38). The first, second, and third groups were injected with Free TanIIA, TanIIA-NSVs, and Gal-pH-TanIIA-NSVs at an equivalent tanshinone IIA dose of 10 mg/kg *via* tail vein, respectively. Orbital blood was taken at 0.083, 0.25, 0.5, 1, 2, 4, 6, 8, 12, and 24 h after administration. Blood was transferred to a centrifuge tube coated with heparin sodium and centrifuged at 9391.2×*g* for 5 min to prepare plasma that was stored at −20°C (39).

Twenty-seven male SD rats were randomly divided into three groups and administered in the same way. Rats were sacrificed after 0.083, 4, and 12 h, respectively, and heart, liver, spleen, lung, and kidney tissues were collected immediately. Any residual blood on the tissue was washed with precooled normal saline, and the washed tissue was blotted up with filter paper and weighed. The tissue was then homogenized in 5 times the mass of precooled normal saline. Homogenized tissue samples were centrifuged at 9391.2×*g* for 5 min to separate the tissue homogenate and were stored at −20°C (39).

After treatment of plasma and tissue homogenate, the obtained supernatant was concentrated by nitrogen blowing and determined by HPLC.

### *In Vivo* Pharmacodynamics Evaluation

An AKT/c-Met-induced HCC mouse model was established by the method of hydrodynamic transfection (40, 41). This is a primary HCC mouse (FVB/N) model triggered by co-overexpression of AKT (also known as protein kinase B) and c-Met (cellular-mesenchymal to epithelial transition factor) proto-oncogenes to evaluate the anti-tumor activity of Gal-pH-TanIIA-NSVs. A total of 2 mL saline solution containing AKT, c-Met, and SB plasmids (mass ratio: 12.5:12.5:1) was injected into wild-type FVB/N mice via tail vein injection. A total of 2 mL of saline solution containing AKT, c-Met, and SB plasmids (mass ratio: 12.5:12.5:1) was injected into wild-type FVB/N mice via tail vein injection. Transfected mice were randomly divided into four groups (six in each group) and fed a standard diet for 3 weeks. Six untransfected mice served as the wild-type group (WT) and were fed the same standard diet. Four weeks later, approximately 0.2 mL of PBS, Free TanIIA, TanIIA-NSVs and Gal-pH-TanIIA-NSVs (equivalent dose of tanshinone IIA is 2 mg/kg) was injected into the transfected mice via the tail vein, once every 2 days for 2 weeks. The weight of all mice was recorded before the first injection and the second day after each injection. After two weeks of administration, mice were sacrificed and their liver tissues were excised. The excised liver tissue was washed and dried before weighing. A portion of the liver tissue was fixed with 4% paraformaldehyde and stained with hematoxylin-eosin (H&E).

### Statistical Methods

All experimental data are presented as the mean ± standard deviation (M ± SD). According to the *P* value obtained by the significance test, *P* < 0.05 was considered statistically significant. Prism 6 software (GraphPad, USA) was used to perform the *t*-test for comparison between two groups and analysis of variance (ANOVA) for comparison among multiple groups.

## RESULTS

### Characterization of Targeted Niosomes Loaded with Tanshinone IIA

**Fig. 1:**
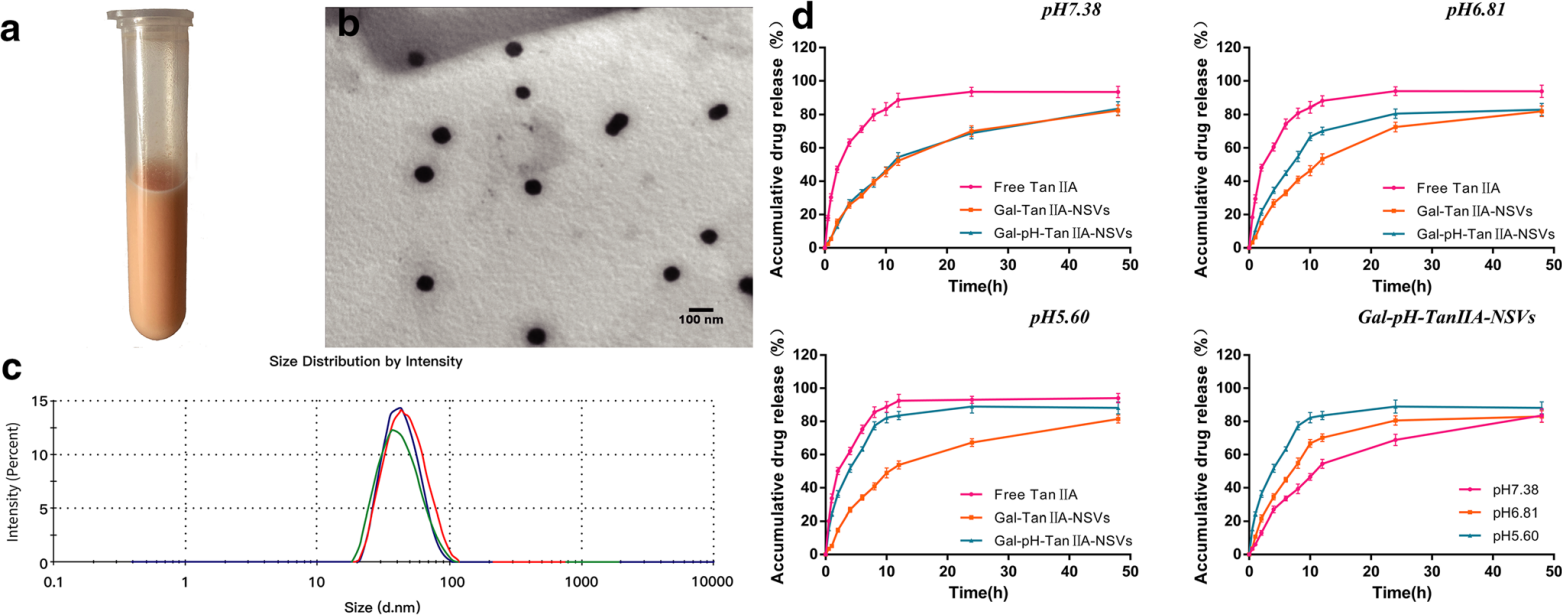
Appearance (**a**), TEM images (**b**), and particle size distribution (**c**) of targeted niosomes loaded with tanshinone IIA. Release curves of free drugs (Free-TanIIA), plain vesicles (Gal-TanIIA-NSVs), and pH-sensitive vesicles (Gal-pH-TanIIA-NSVs) at a different pH, along with the release curves of pH-sensitive vesicles in different acidity media (**d**)

Particle size and zeta potential were determined for four types of niosomes loaded with tanshinone IIA, which were indistinguishable in appearance (Fig. 1a). The results (Table I) showed that there was no significant difference in the encapsulation efficiency of the four types of vesicles, and modification with galactosylated stearate slightly increased the particle size of the vesicles; however, the zeta potential was not significantly altered, and the addition of CHEMS significantly decreased the particle size, while the absolute value of the zeta potential increased. It may be that the glycosyl groups exposed in the hydrophilic region of the outer layer increase the surface area of the vesicles leading to an increase in particle size, whereas galactosylated stearate contains no ionizable groups and thus has little effect on the surface charge of the vesicles. The lamellar organization of CHEMS in neutral and alkaline aqueous media reduces the fluidity of the bilayer and makes the bilayers more “compact” or “thicker,” whereas the cholesterol forms as monohydrated crystals in an aqueous environment, thus the size of the vesicles after addition of CHEMS decreases and the stability increases. The particle size of the final form of the preparation (Gal-pH-TanIIA-NSVs) was about 53.72 nm and was mainly distributed in the range of 20–100 nm (Fig. 1c), and the zeta potential was −28.31 mV (Table I). TEM observation showed that the appearance of Gal-pH-TanIIA-NSVs was round and smooth, and the distribution was uniform (Fig. 1b). The particle size was similar to that determined by nanoparticle size analyzer. Moreover, the encapsulation efficiency of Gal-pH-TanIIA-NSVs reached 84.70%, and the leakage was only 3.43% after 60 days of storage at 2–8°C.

**Table I:** Characteristics of Targeted Niosomes Loaded with Tanshinone IIA

Groups	Average size (nm)	PDI	Zeta potential (mV)	EE (%)
TanIIA-NSVs	66.72 ± 1.21	0.183 ± 0.03	−20.75 ± 0.99	84.21 ± 1.23
Gal-TanIIA-NSVs	69.04 ± 2.07	0.154 ± 0.05	−21.21 ± 1.08	83.97 ± 0.97
pH-TanIIA-NSVs	51.66 ± 1.28	0.172 ± 0.04	−28.13 ± 1.32	85.04 ± 2.05
Gal-pH-TanIIA-NSVs	53.72 ± 0.91	0.156 ± 0.01	−28.31 ± 1.44	84.70 ± 0.47

*PDI*, polymer dispersity index (is used to describe the molecular weight distribution of polymers); *EE*, encapsulation efficiency

### *In Vitro* Release of Targeted Niosomes Loaded with Tanshinone IIA

*In vitro* release of targeted niosomes loaded with tanshinone IIA was determined by the dialysis bag method. At pH 7.38, plain vesicles and pH-sensitive vesicles showed a very similar release, and the accumulative release percentage was not more than 50% until 12 h. Throughout the process, drug-loaded vesicles showed significantly sustained release characteristics relative to free drugs. At pH 6.81, the release process of plain vesicles was similar to that at pH 7.38. The release of pH-sensitive vesicles was slightly faster than that of plain vesicles, and the accumulative release percentage at 8 h exceeded 50% (Fig. 1d).

The release rate of pH-sensitive vesicles was significantly faster in an acidic environment compared to an alkaline environment, mainly because CHEMS, an acid-sensitive excipient, was added to the formulation for the preparation of pH-sensitive vesicles. The acidic environment will lead to protonation of the carboxyl group of CHEMS, and change niosomes from liquid crystals to liquid, thereby reducing the zeta potential and the stability of the bimolecular layer. Destabilization of the bimolecular layer induced the recombination of amphiphilic molecules, leading to aggregation and rupture of vesicles, and finally, release of the drug encapsulated inside (42, 43).

### Qualitative Investigation of Targeting *In Vitro* by Inverted Fluorescence Microscopy

**Fig. 2:**
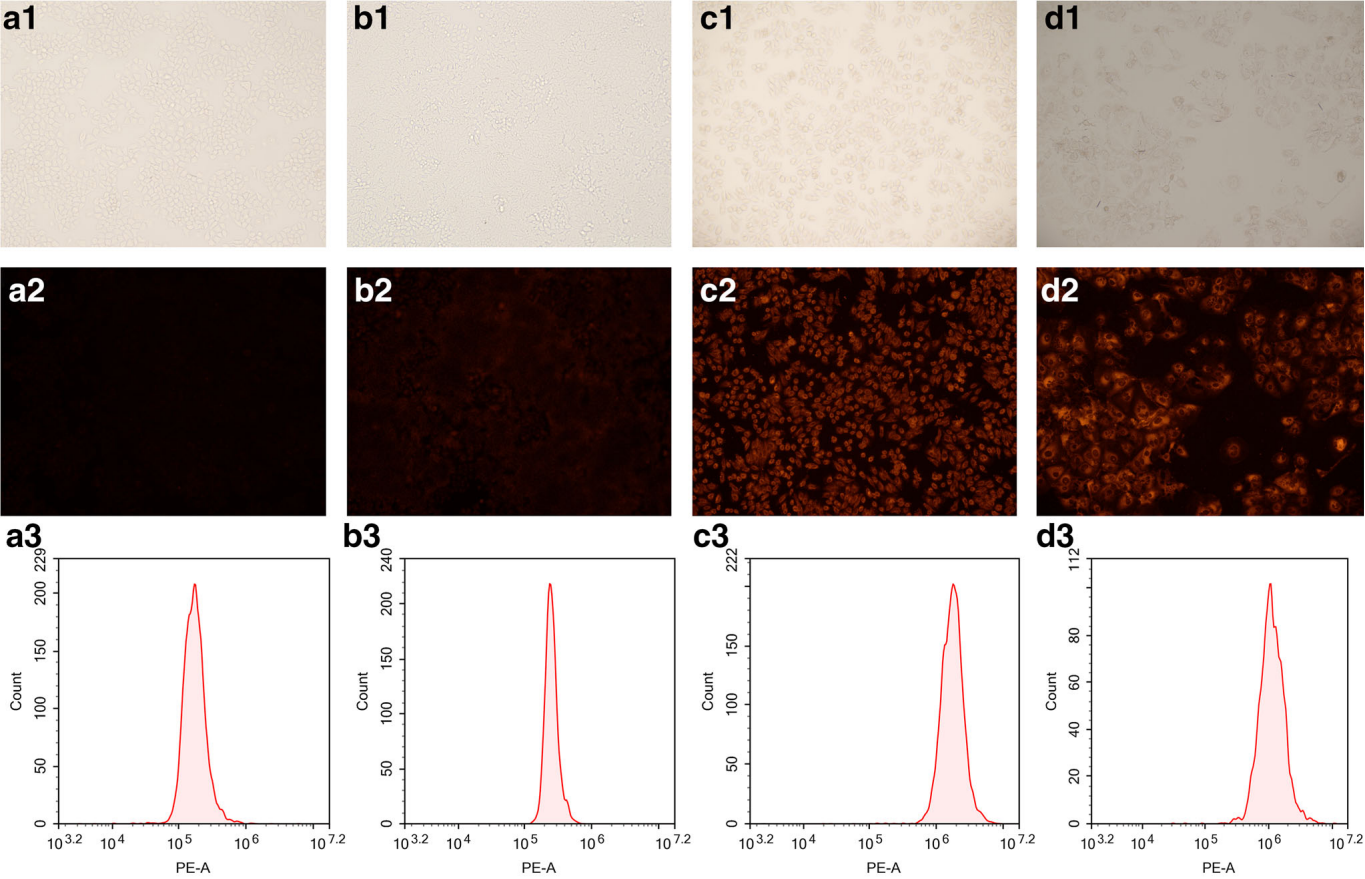
Mean fluorescence intensity (3) of A2780 (**a**), HCT8 (**b**), HepG2 (**c**), and Huh7 (**d**) cells treated with 10% Gal-pH-NR-NSVs, and images of bright fields (1) and fluorescent fields (2) were taken by inverted fluorescence microscopy

Qualitative analysis showed that A2780 cells and HCT8 cells treated with 10% Gal-pH-NR-NSVs showed very faint fluorescence characteristics. In contrast, hepatoma cells treated with the same conditions showed intense fluorescence characteristics, which indicated that niosomes modified with galactosylated stearate had better targeting ability to hepatoma cells, and did not easily target A2780 and HCT8 cells without ASGPR expression (Fig. 2).

### Quantitative Investigation of Targeting *In Vitro* by Flow Cytometry

**Fig. 3:**
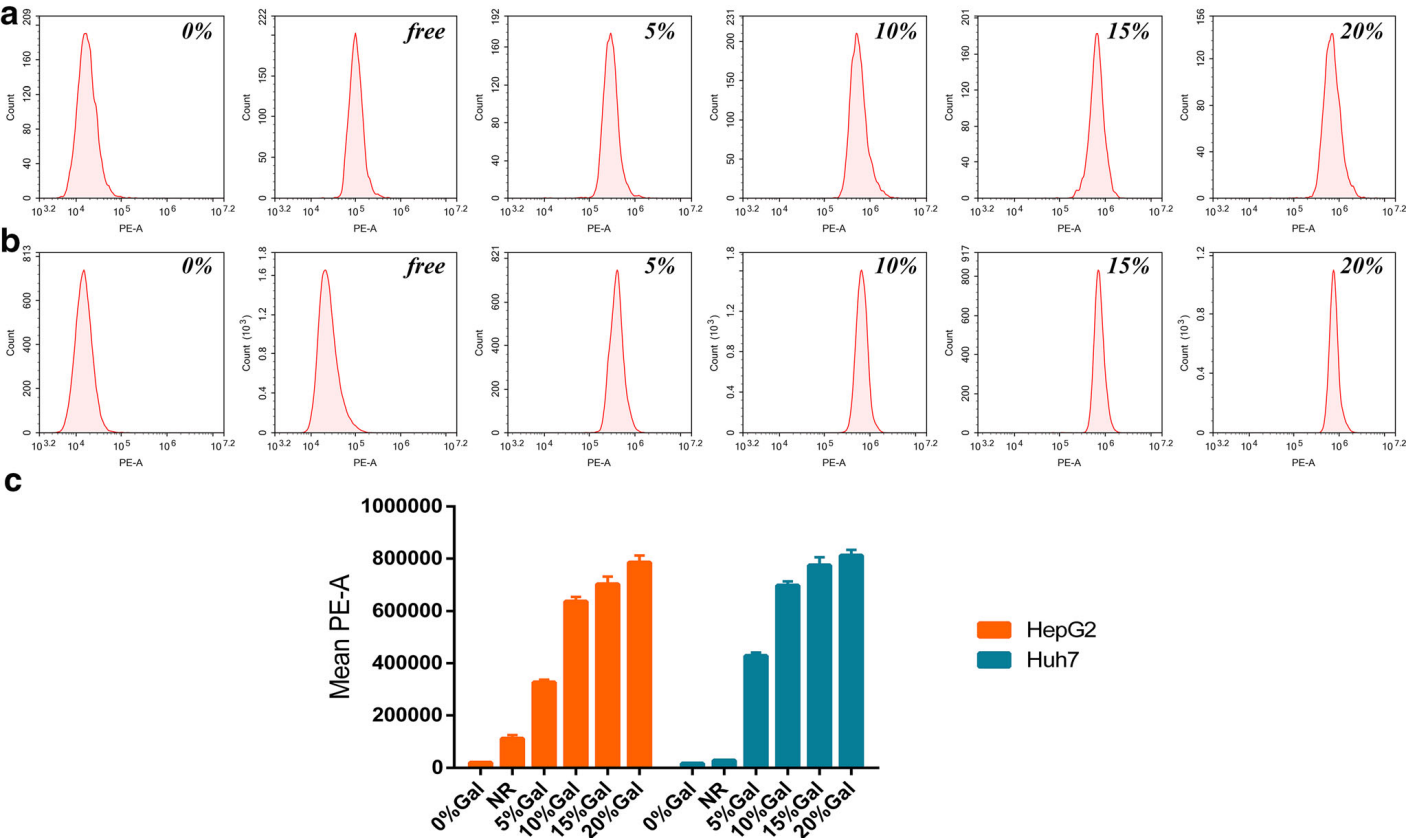
The mean fluorescence intensity (**c**) of HepG2 (**a**) and Huh7 (**b**) cells treated with 0% Gal-pH-NR-NSVs, Free NR, 5% Gal-pH-NR-NSVs, 10% Gal-pH-NR-NSVs, 15% Gal-pH-NR-NSVs, and 20% Gal-pH-NR-NSVs, respectively

Compared with other tumor cells, the fluorescence intensity results determined by flow cytometry indicated that galactose-modified niosomes showed high fluorescence intensity in hepatoma cells, which was consistent with the qualitative analysis results. The fluorescence intensity of cells was enhanced by increasing the dosage of galactosylated stearate and was higher than that of cells treated with free Nile Red. These findings further demonstrated that galactosylated stearate modified niosomes could better target hepatoma cells. When the dosage of galactosylated stearate was more than 10%, the increasing trend of fluorescence intensity was slower than before (Fig. 3). Other studies have shown that free galactose ligands can compete with galactosylated macromolecules to bind to ASGPR on hepatocytes. Therefore, the dosage of galactosylated stearate should not be too large to avoid competitive binding of free galactosylated stearate and galactose-modified niosomes to hepatocytes, which can inhibit the uptake of niosomes by cells, therefore, the dosage of galactosylated stearate in the prescription is 10%. In other words, the molar ratio of tanshinone IIA to galactosylated stearate is 1:6. The reason why galactose can increase the targeting of vesicles is that ASGPR is present on the surface of mammalian hepatocytes (tumor cells also express ASGPR) and is characterized by high tissue specificity, high affinity, and high capacity. ASGPR can recognize the glycoprotein-containing galactose at the end and induce receptor-mediated endocytosis of hepatocytes, internalizing the glycoprotein inside the cells (44), while galactosylated niosomes just meet this characteristic.

### Evaluation of Anticancer Activity *In Vitro* Via the CCK8 Assay

**Fig. 4:**
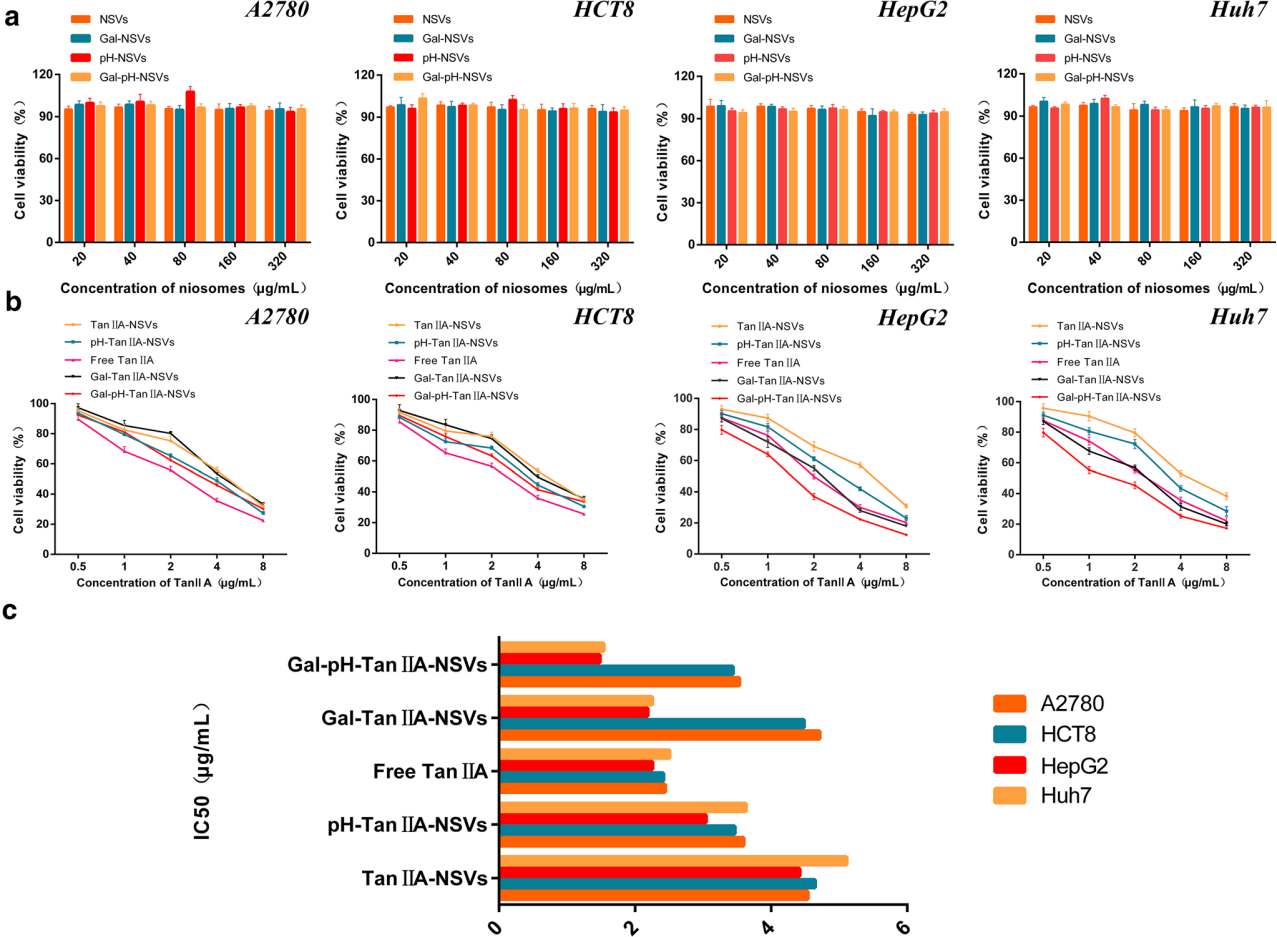
Cytotoxicity test results of A2780, HCT8, HepG2, and Huh7 cells treated with different concentrations of vesicle materials (**a**) and the cell viability of A2780, HCT8, HepG2, and Huh7 cells treated with tanshinone IIA-loaded niosomes and free tanshinone IIA at different concentrations of tanshinone IIA (**b**). IC_50_ values of tanshinone IIA-loaded niosomes and free tanshinone after treatment of A2780, HCT8, HepG2, and Huh7 cells (**c**)

Blank vesicles showed no cytotoxicity to A2780, HCT8, HepG2, and Huh7 cells with a cell viability greater than 93%, 93%, 92%, and 95% at a concentration of 320 μg/mL, respectively (Fig. 4a). A2780, HCT8, HepG2, and Huh7 cell inhibition assays showed that both free tanshinone IIA and tanshinone IIA-loaded niosomes significantly inhibited the survival of the four types of cells in a concentration-dependent manner (45) (Fig. 4b). With the IC50 as an indicator of anti-tumor activity, the anti-tumor activity of different tanshinone IIA preparations in A2780 and HCT8 cells was as follows: Free TanIIA (2.442, 2.416 μg/mL) > pH-TanIIA-NSVs (3.596, 3.464 μg/mL) ≈ Gal-pH-TanIIA-NSVs (3.532, 3.437 μg/mL) > TanIIA-NSVs (4.540, 4.646 μg/mL) ≈ Gal-TanIIA-NSVs (4.710, 4.480 μg/mL). The anti-tumor activity of different tanshinone IIA preparations in HepG2 and Huh7 cells was as follows: Gal-pH-TanIIA-NSVs (1.481, 1.539 μg/mL) > Gal-TanIIA-NSVs (2.185, 2.252 μg/mL) ≈ Free TanIIA (2.256, 2.505 μg/mL) > pH-TanIIA-NSVs (3.042, 3.627 μg/mL) > TanIIA-NSVs (4.419, 5.108 μg/mL) (Fig. 4c).

The inhibitory effects of free drugs on four types of tumor cells were comparable, and free drugs had the strongest antitumor activity on non-hepatoma cells, while Gal-pH-TanIIA-NSVs had the most powerful anti-tumor activity on hepatoma cells, which again confirmed that galactose-modified vesicles had hepatic targeting characteristics. These results are attributed to the fact that galactose-modified pH-sensitive niosomes have proper targeting and pH sensitivity to hepatoma cells and can be taken up by hepatoma cells in large quantities and have high intracellular release efficiency. The anti-tumor activity of TanIIA-NSVs was the weakest in both hepatoma cells and non-hepatoma cells, mainly because Tan IIA was encapsulated in niosomes and had a sustained release effect, resulting in smaller amounts of drug acting on cells in a limited time, resulting in a higher IC50 value. The addition of pH-sensitive excipients accelerated the drug release in tumor cells, so that tanshinone IIA-loaded niosomes containing CHEMS had a stronger anti-tumor activity than TanIIA-NSVs in all cells.

### Apoptosis Analysis of HepG2 Cells

**Fig. 5:**
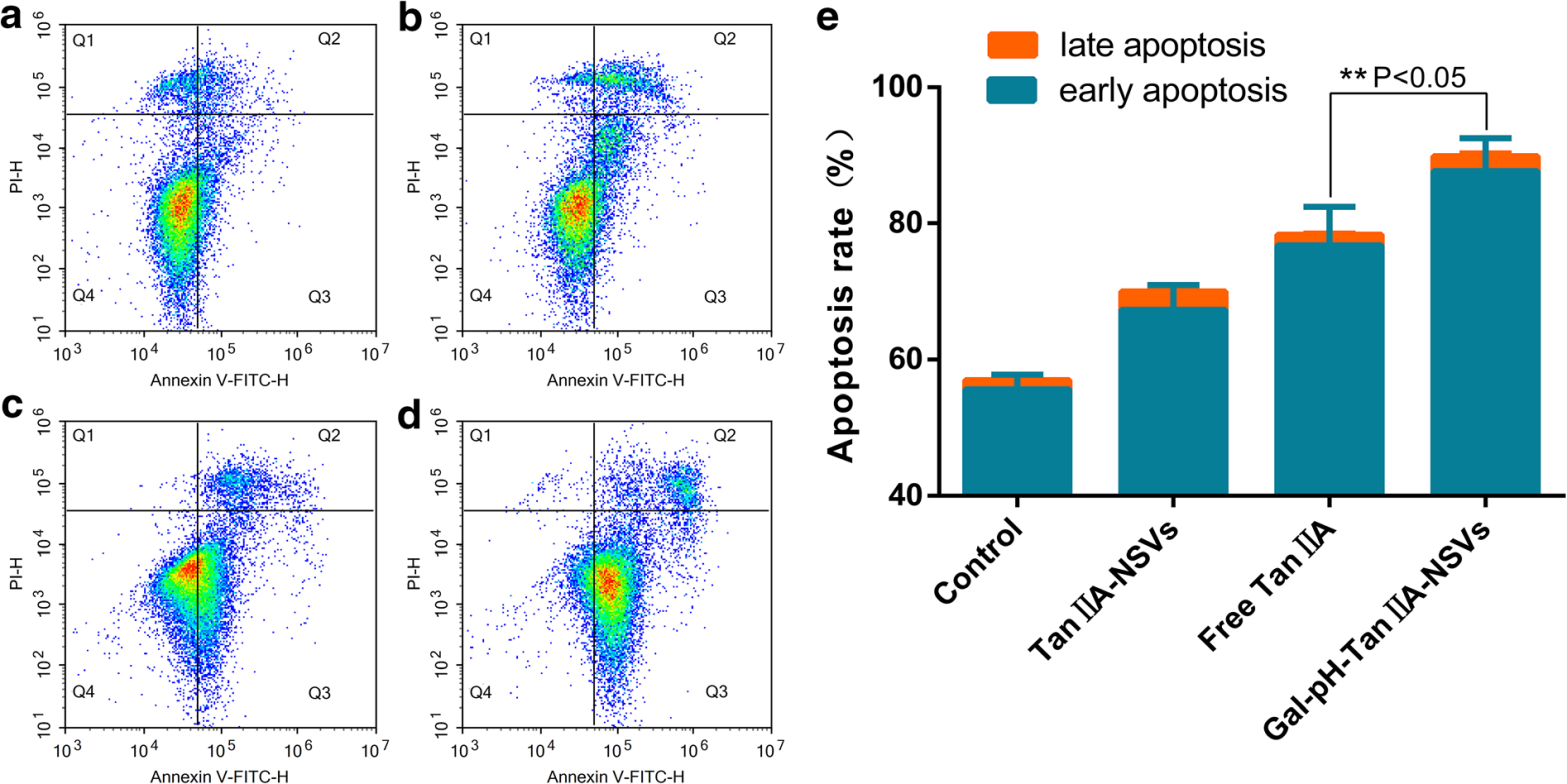
Apoptosis distribution and apoptotic rate (**e**) of HepG2 cells treated with PBS (**a**), Tan IIA-NSVs (**b**), Free Tan IIA (**c**), and Gal-pH-Tan IIA-NSVs (**d**), respectively

Early apoptosis and late apoptosis were combined to calculate the apoptotic rate. The apoptotic rates of the control, TanIIA-NSVs, Free TanIIA, and Gal-pH-TanIIA-NSV groups were 57.33%, 70.46%, 78.34%, and 89.89%, respectively (Fig. 5). When compared with the free drug group, the apoptotic rate of the plain vesicle group was lower, probably because tanshinone IIA encapsulated in the plain vesicle had a sustained release effect, smaller amounts of drug acted on the cells within a limited time, resulting in a lower apoptotic rate than that of the free drug group, which was consistent with the results of the CCK8 assay. The apoptotic rate in the targeted vesicle group was significantly higher than that in the free drug group. Combined, these results indicated targeted niosomes loaded with tanshinone IIA significantly better induced apoptosis of HepG2 cells than plain vesicles loaded with tanshinone IIA and free tanshinone IIA at a dosage of 5 μg/mL.

### Cycle Analysis of HepG2 Cells

**Fig. 6:**
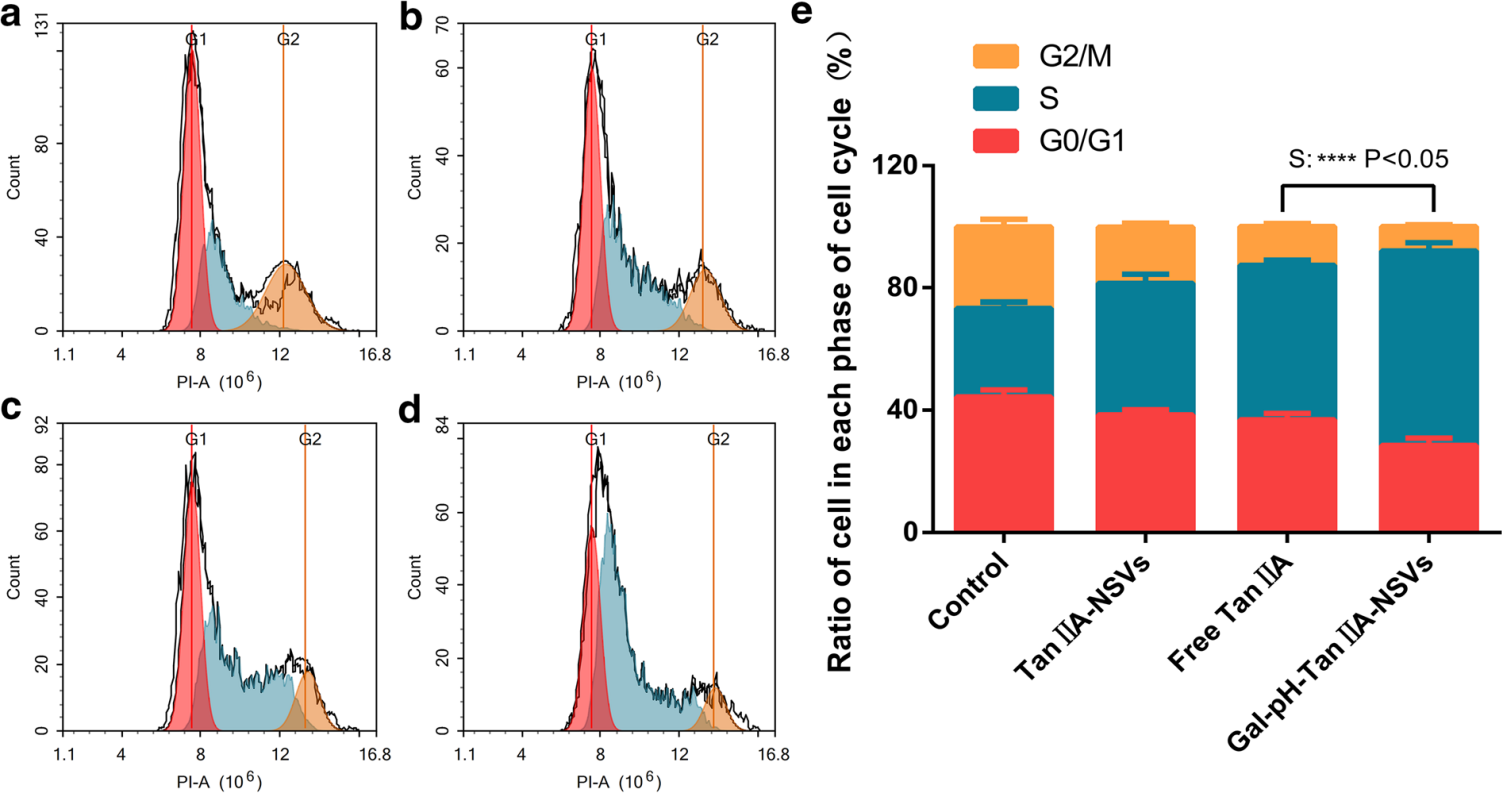
Cell cycle distribution and cell cycle ratio (**e**) of HepG2 cells treated with PBS (**a**), Tan IIA-NSVs (**b**), Free Tan IIA (**c**), and Gal-pH-Tan IIA-NSVs (**d**), respectively

The cell cycle results indicated that the S phase of the control, TanIIA-NSVs, Free TanIIA, and Gal-pH-TanIIA-NSV groups were 28.98%, 42.94%, 50.53%, and 63.64% (Fig. 6), respectively. Some studies have reported that tanshinone IIA can block the S-phase of HepG2 cells, thereby resulting in an increased distribution of the S-phase. The above-mentioned data showed that Gal-pH-TanIIA-NSVs could induce ASGPR-mediated endocytosis of HepG2 cells and increase the uptake of tanshinone IIA by HepG2 cells, thus increasing the potent effect of blocking the S-phase. HepG2 cells treated with PBS (control), TanIIA-NSVs, Free TanIIA, and Gal-pH-TanIIA-NSVs, respectively, showed a decline in the distribution of the G0/G1-phase, but a significant increase in the distribution of the S-phase, resulting in a reducing trend of the G2/M-phase. Comparing all groups, the Gal-pH-TanIIA-NSV group had a potent blocking effect on the S-phase, resulting in a minimal number of cells in the G2/M-phase. Thus, we hypothesized that targeted niosomes loaded with tanshinone IIA act as effective proliferation inhibitors of HepG2 cells.

### Pharmacokinetics and Biodistribution of Targeted Niosomes Loaded with Tanshinone IIA

**Fig. 7:**
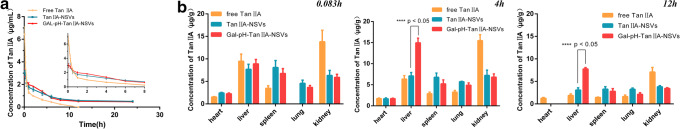
Pharmacokinetic profiles of Tan IIA in SD rats after intravenous administration of free Tan IIA or Tan IIA-loaded niosomes at an equivalent dose of 10 mg Tan IIA/kg. Tan IIA was extracted from plasma and measured by HPLC. The inset magnifies the pharmacokinetic profiles between 0.083 and 6 h (**a**). Biodistribution of tanshinone IIA at 0.083, 4, and 12 h after administration (**b**)

The results of pharmacokinetic experiments indicated that the plasma concentration of the free drug group decreased significantly after administration and could not be detected by HPLC 12 h after injection. However, tanshinone IIA encapsulated in niosomes still had a detectable concentration at 24 h (Fig. 7a), indicating that it had a longer blood circulation time. Compared with free drugs, tanshinone IIA encapsulated by niosomes had a larger area under the curve (AUC), a longer mean residence time (MRT), and a longer half-life (*T*_1/2_) and theoretically had a better systemic drug effect (Table II). A longer MRT and a lower clearance rate (CL) prolonged the blood circulation time of the drug, resulting in a larger AUC (Table II). It is interesting to note that although the Gal-pH-TanIIA-NSV group had a longer MRT and a lower CL than the Free Tan IIA group, their apparent volume of distribution (Vd) was smaller (Table II). The targeted niosomes loaded with tanshinone IIA had a hepatic targeting effect, leading to drug aggregation at the liver site, which interfered with drug distribution to the whole body via blood circulation, thereby resulting in the smaller volume of distribution, which also explained that the Gal-pH-TanIIA-NSV group had a higher CL and a shorter MRT than the TanIIA-NSV group (Table II).

**Table II:** Pharmacokinetic Parameters.

Parameters	Groups		
	Free TanIIA	TanIIA-NSVs	Gal-pH-TanIIA-NSVs
AUC_0–t_ ((μg/mL)·h)	7.70 ± 1.51	19.65 ± 3.06	19.31 ± 3.19****
AUC_0–∞_ ((μg/mL)·h)	8.81 ± 1.65	28.23 ± 6.12	25.03 ± 2.96****
MRT_0–t_ (h)	2.23 ± 0.20	8.83 ± 0.61	8.27 ± 0.70****
MRT_0–∞_ (h)	3.53 ± 0.63	18.56 ± 1.28	14.64 ± 0.87****
*T*_1/2_ (h)	3.09 ± 0.59	11.66 ± 1.08	8.43 ± 0.54****
Vd (mg)/(μg/mL)	1.26 ± 0.09	1.49 ± 0.24	1.22 ± 0.10
CL (mg)/(μg/mL)/h	0.284 ± 0.026	0.089 ± 0.021	0.100 ± 0.024

****p < 0.05 vs. Free TanII. *AUC*, area under the curve; *MRT*, mean residence time; *T*_*1/2*_, half-life; *Vd*, apparent volume of distribution; *CL*, clearance rate

The results of the biodistribution experiments showed that free tanshinone IIA in kidney tissue maintained relatively high drug levels at all time periods tested after administration, which indicated that free tanshinone IIA experienced a faster drug elimination process *in vivo*. At 4 h and 12 h after administration, the accumulation of tanshinone IIA encapsulated by niosomes in the liver was more obvious (concentration of TanIIA is higher) than that of free tanshinone IIA, especially Gal-pH-TanIIA-NSVs. The niosomes modified by galactose had a liver targeting effect and showed significant affinity to the liver site. We also found that the content of tanshinone IIA encapsulated by niosomes in the spleen was higher than that of free tanshinone IIA, probably because the spleen, similar to the liver, has a well-developed reticuloendothelial system, which can selectively ingest niosomes, liposomes, and other nanoparticles. Taken together, these results indicated that Gal-pH-TanIIA-NSVs effectively prolonged the retention time of tanshinone IIA in the liver and significantly increased the biodistribution of tanshinone IIA in the liver (Fig. 7b).

### *In Vivo* Anticancer Activity Study

**Fig. 8:**
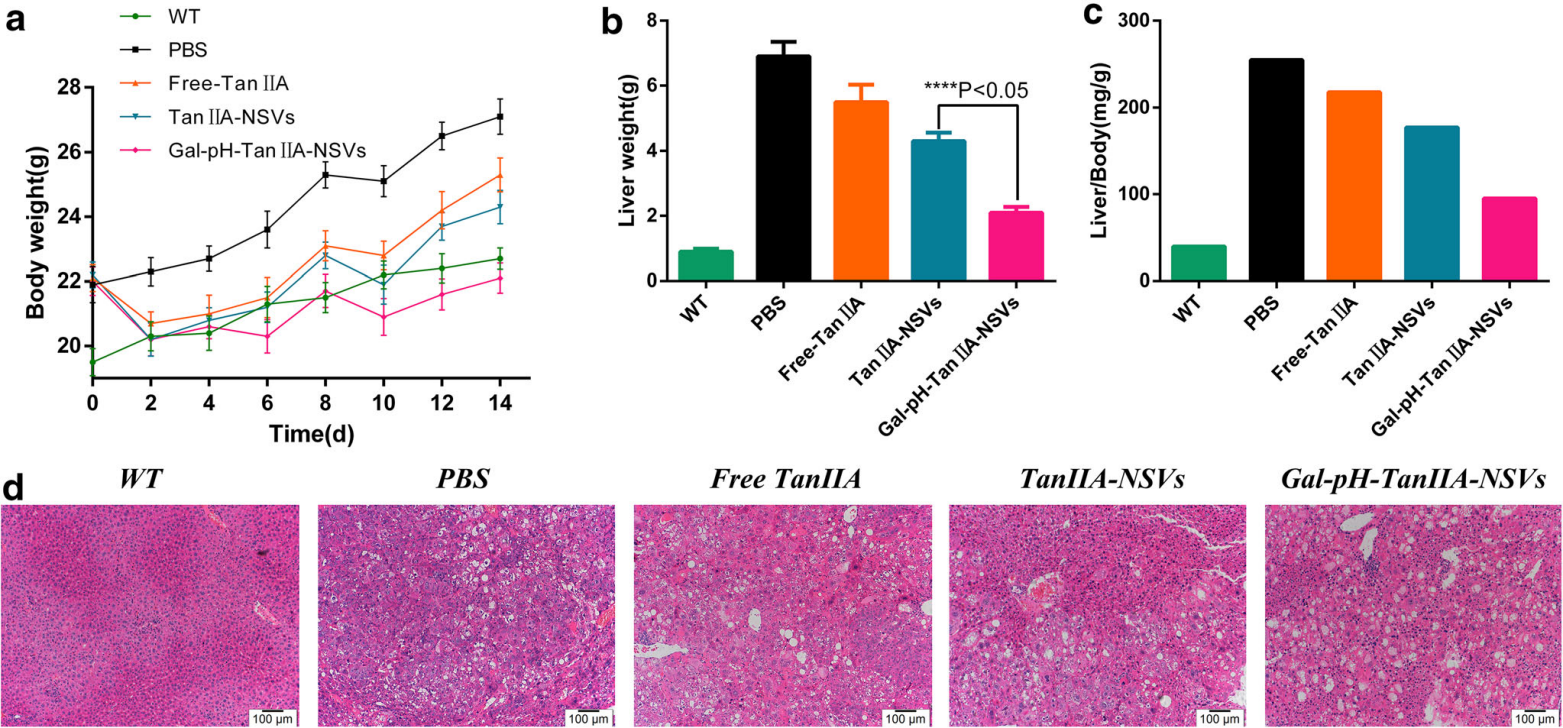
The body weight of mice increased with days after administration (**a**). Liver weight (**b**) and the liver/body weight ratio (**c**) of mice after treatment with different formulations for 2 weeks. H&E staining of liver tissue of mice in each group (**d**)

A rapid HCC mouse model established by hydrodynamic transfection of AKT and c-Met plasmid was used to evaluate the anti-tumor activity of Gal-pH-TanIIA-NSVs. The weight gain of PBS-treated transfected mice (PBS group) was significantly greater compared to that of untransfected mice (WT group). Mice in the preparation group (Free TanIIA, TanIIA-NSVs, Gal-pH-TanIIA-NSVs) showed some weight loss at the initial stage of administration, however, with the continuation of experiments, the weight gradually increased (Fig. 8a). It is possible that the initial inadaptation of mice to the tail vein injection affected their appetite and the anti-hepatoma effect of the drugs inhibited the increase of liver weight, eventually leading to weight loss. As the experiment continued, the physiological function of mice gradually adapted to the current situation, and the body weight of mice increased; however, the body weight of mice in each group eventually changed to different degrees due to the differences in drug formulations. Among all groups, mice in the Gal-pH-TanIIA-NSV group showed the lowest weight gain, while mice in the PBS group showed the most weight gain. The final liver weight data (Fig. 8b) showed that the most important reason for weight gain in the PBS group was the increase in liver weight. The liver weight of mice in the Gal-pH-TanIIA-NSV group was about 1/3 of that of mice in the Free-TanIIA group and 1/2 of that of mice in the TanIIA-NSV group. The above findings were confirmed by the data of the liver weight to body weight ratio (Fig. 8c).

The results of H&E staining of the liver of mice in each group showed that the structure of liver tissue in the WT group was clear, the nuclei were evenly distributed, and the cytoplasm was abundant. In the PBS group, the liver tissue structure was destroyed, significant vacuolar degeneration was apparent, nuclei were enlarged and scattered, and the cytoplasm almost disappeared. In the preparation group, liver tissue lesions were alleviated, the cell structure was clear, vacuolar degeneration was reduced, nuclei were smaller, multinucleated cells were significantly reduced, and hepatocytes were filled with a small amount of cytoplasm. In the Gal-pH-TanIIA-NSV group, the improvement was most obvious, and normal tissue was visible near tumor lesions (Fig. 8d).

## DISCUSSION

Due to the generally serious toxic and side effects of chemotherapy, the bioactive components in natural herb medicine are considered potential candidates for the treatment of cancer. The bioactive compound of tanshinone IIA in natural medicine *Salvia miltiorrhiza* Bge can act on human hepatoma cells and induce apoptosis (46), inhibit cell proliferation, regulate protein expression in HCC *in vitro*, inhibit the activity of fatty acid synthase (47), and significantly reduce the phosphorylation of p38-MAPK and AKT in human hepatoma cells (48). These data suggest that tanshinone IIA is a potential candidate drug for the treatment of HCC. However, tanshinone IIA is insoluble in water and has a short half-life, which greatly limits its clinical application. Therefore, tanshinone IIA was encapsulated in niosomes to overcome these shortcomings and improve its therapeutic efficiency by further modification of the vesicles.

ASGPR is a C-type lectin expressed on sinus of gaps (49). Because of its presence on the surface of mammalian hepatocytes, it is characterized by high tissue specificity, high affinity, and high capacity (50). The presence of ASGPR provides galactosylated macromolecules with hepatic targeting characteristics. Therefore, galactosylation of nanoparticles drug delivery system (NDDS) has become a hot spot in the research of targeting drug delivery system (TDDS). The traditional way to galactosylate vesicles is to link glycoproteins on the surface of vesicles. Still, this exogenous biomacromolecule targeting ligand has a high molecular weight, resulting in an excessive particle size of the prepared vesicles, which is not conducive to targeting hepatocytes. In addition, it also elicits a potential immune response of the body (51). Therefore, we aimed to synthesize a galactosylated amphiphilic lipid, galactosylated stearate. In the preparation process, galactosylated stearate was similar to that involved in the formation of the bimolecular layer, thus enabling ordered arrangement in the bimolecular layer and exposing the water-soluble head of galactose to the vesicle surface, so that the modified vesicles are recognized by ASGPR. In doing so, some of the shortcomings of traditional methods were prevented. At present, CHEMS is a commonly used excipient in the preparation of acid-sensitive preparations. It not only stabilizes the bimolecular layer like cholesterol, but also enables the rapid release of the preparations in an acidic environment. In this study, the dialysis bag method was used to simulate the drug release process *in vivo*, and the acid sensitivity of the preparation was adjusted by changing the ratio of cholesterol to CHEMS to identify the most suitable formulation for the intracellular release in hepatoma cells.

It has been reported that nanoparticles with a particle size of 100–200 nm are quickly cleared from the blood by macrophages in the reticuloendothelial system after entering the blood circulation, and finally reach the lysosome of Kupffer cells in the liver. In comparison, nanoparticles with a particle size of 50–100 nm can enter hepatocytes, and target drugs to the liver. Nanoparticles with a particle size of less than 50 nm can be transported through liver endothelial cells or through lymphatic system to the spleen or bone marrow (52). According to the enhanced permeability and retention (EPR) effect, because of the large gap between vascular endothelial cells in tumor tissues, particles with particle size below 100 nm are effortlessly exuded and retained in tumor tissues (53). Therefore, the preparation of vesicles with particle size between 50 and 100 nm has become an essential factor affecting the performance of this preparation. In this study, considering the low toxicity and volatility of ethanol and the fact that Span80 is an oily liquid at room temperature, the ethanol injection method was chosen to prepare targeted niosomes. Technological factors were optimized, and a liposome extruder was utilized, to ensure that the final prepared vesicles have a particle size of around 53 nm.

In this study, *in vitro* release experiments and *in vitro* and *in vivo* pharmacodynamics experiments were carried out. All experimental results indicated that targeted niosomes containing tanshinone IIA could recognize hepatoma cells via the mediation of galactose ligand and improve the drug uptake by hepatoma cells. The application of pH-sensitive excipients accelerates the drug release in tumor cells. Compared with free drugs, tanshinone IIA encapsulated in targeted niosomes significantly increased the ability to induce apoptosis and inhibit proliferation of hepatoma cells, and showed the most effective anti-tumor efficacy. In addition, pharmacokinetic experiments showed that tanshinone IIA encapsulated in targeted niosomes had a longer half-life and a lower clearance rate than free drugs; however, its apparent volume of distribution was smaller, suggesting that the tanshinone IIA encapsulated in targeted niosomes had an increased liver distribution relative to free drugs. Biodistribution studies further illustrated the *in vivo* targeting effect of this preparation.

## CONCLUSION

A novel hepatic targeted niosome delivery system featuring specific recognition of hepatoma cells and rapid drug release in tumor cells was developed to deliver the natural bioactive compound tanshinone IIA to hepatoma cells. This multifunctional vesicle has unique advantages:

I. Targeted niosomes have high biocompatibility and very low cytotoxicity.
II. Galactose-modified niosomes can be recognized by the liver and enrich drug release in hepatic tumors.
III. The application of CHEMS accelerates drug release in tumor cells.
IV. Pharmacodynamic experiments *in vivo* and *in vitro* confirm that the preparation indeed has more powerful anti-tumor activity.
V. Delivery of tanshinone IIA using this delivery system exhibits a longer half-life and an increased liver distribution *in vivo*.

This preparation showed stronger antitumor activity in both cell experiments and an AKT/c-Met-induced HCC mouse model, which also indicated that this drug delivery system is an effective carrier for the delivery of anti-hepatoma drugs.
